# Supplementary material for: Flexible heat-flow sensing sheets based on the longitudinal spin Seebeck effect using one-dimensional spin-current conducting films
Source: Sci Rep. 2016 Mar 15;6:23114. doi: 10.1038/srep23114 (PMC4791552; doi:10.1038/srep23114)
Supplement: Supplementary Information [file srep23114-s1.pdf]

# Supplementary Information:
# Flexible heat-flow sensing sheets based on the longitudinal spin Seebeck effect using one-dimensional spin-current conducting films

Akihiro Kirihara[1,2*], Koichi Kondo[3], Masahiko Ishida[1,2], Kazuki Ihara[1,2], Yuma Iwasaki[1], Hiroko Someya[1,2], Asuka Matsuba[1], Ken-ichi Uchida[4,5], Eiji Saitoh[2,4,6,7], Naoharu Yamamoto[3], Shigeru Kohmoto[1] and Tomoo Murakami[1]

[1]*Smart Energy Research Laboratories, NEC Corporation, Tsukuba, 305-8501, Japan*

[2]*Spin Quantum Rectification Project, ERATO, Japan Science and Technology Agency, Sendai, 980-8577, Japan*

[3]*NEC TOKIN Corporation, Sendai, 982-8510, Japan*

[4]*Institute for Materials Research, Tohoku University, Sendai, 980-8577, Japan*

[5]*PRESTO, Japan Science and Technology Agency, Saitama, 332-0012, Japan*

[6]*WPI, Advanced Institute for Materials Research, Tohoku University, Sendai, 980-8577, Japan*

[7]*Advanced Science Research Center, Japan Atomic Energy Agency, Tokai, 319-1195, Japan*

**TE experiment in TSSE setup** We have performed additional experiments to check whether transverse spin Seebeck effect (TSSE) [1-3] contributes to the TE signal in our devices. The schematics of the experimental setup is shown in Fig S1(a). For the TSSE experiment, we prepared a TE-sheet sample in a similar fashion as described in the main article. In the sample, a $20 \times 5\text{-mm}^2$ Pt strip with a thickness of 5 nm was formed on an edge of $20 \times 20\text{-mm}^2$ ferrite-plated film, as shown in Fig. S1(a). To investigate the TSSE, output voltage $V$ along the $y$-direction between two ends of the Pt strip was measured, when a temperature difference $\Delta T$ was applied in the $x$-direction. To magnetize the ferrite-plated film, an external magnetic field $H$ was also applied in the $x$-direction. If the TE-sheet sample exhibits the TSSE, output voltage is expected to occur in the $y$-direction. Figure S1(b) shows the measured $V$ as a function of $H$ when $\Delta T = 1.1\text{K}$ was applied to the sample, where no output signal was clearly observed. The result suggests that our TE sheet with a ferrite-plated film having one-dimensional spin-current conducting properties does not exhibit any TE voltage originating from the TSSE, since a transverse spin current is effectively blocked by its columnar crystalline structure.

**LSSE-based TE sheets with different metallic films** To gain further insights into the TE mechanism, we also prepared and evaluated TE sheets using different metal-film materials instead of Pt. TE measurements were performed in the same experimental configuration as mentioned above. Figure S2(a) represents the TE voltage from a sample composed of a 10-nm-thick Cu film and a 500-nm- thick $Ni_{0.2}Zn_{0.3}Fe_{2.5}O_4$ film on a polyimide substrate, showing that the output voltage from the

Cu film is negligibly small. This result is consistent with the negligible ISHE in Cu, which has a weak spin-orbit interaction. Figure S2(b) shows the experimental result of a TE sheet in which a W film with a thickness of 5 nm was deposited on the same $Ni_{0.2}Zn_{0.3}Fe_{2.5}O_4$/polyimide substrate. In this case, the clear TE voltage $V$ was observed and its sign was found to be opposite to that of the Pt/ $Ni_{0.2}Zn_{0.3}Fe_{2.5}O_4$ sample (compare Fig. S2(b) with Fig. 2(d) in the main article), which is consistent with the fact that the spin-Hall angle of W has a sign opposite to that of Pt [4,5]. Notably, the heat-flow sensitivity of the W/$Ni_{0.2}Zn_{0.3}Fe_{2.5}O_4$ sensor is $V/q$ = 3.55 nV/(W/m$^2$), a value 3.5-fold greater than that of the Pt/$Ni_{0.2}Zn_{0.3}Fe_{2.5}O_4$, although the W-film resistance between the ends of the sample ($R_W$ = 2.04 kΩ) was an order of magnitude greater than that of the Pt film. This large value suggests that W appears to be a promising material for heat-flow sensing applications.

**Reference**


[1] Uchida, K. et al. Observation of the spin Seebeck effect. *Nature* **455,** 778-781 (2008).

[2] Uchida, K. et al. Spin Seebeck insulator. *Nat. Mater.* **9,** 894-897 (2010).

[3] Jaworski, C. et al. Observation of the spin-Seebeck effect in a ferromagnetic semiconductor. *Nat. Mater.* **9,** 898-903 (2010).

[4] Tanaka, T. et al. Intrinsic spin Hall effect and orbital Hall effect in 4d and 5d transition metals. *Phys. Rev. B* **77,** 165117 (2008).

[5] Ishida, M. et al. Observation of longitudinal spin Seebeck effect with various transition metal films. arXiv:1307.3320.

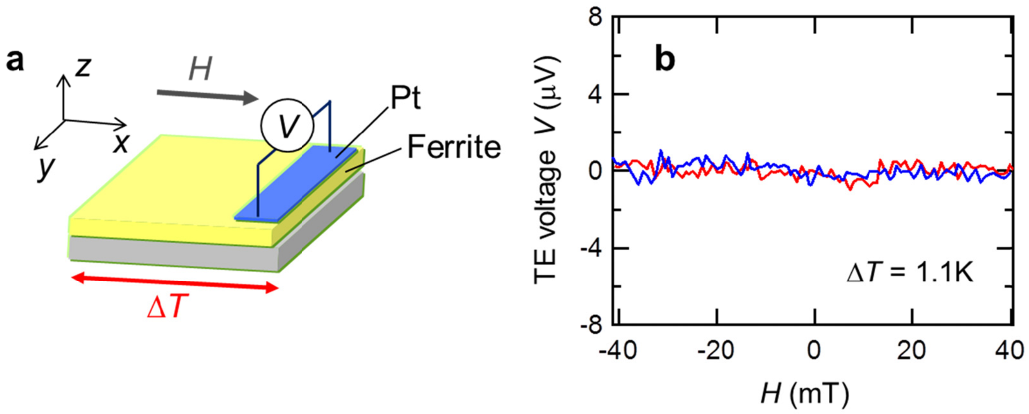


**Figure S1 | TE experiment in transverse-SSE setup.** (a) Experimental set up for checking the transverse SSE. Output voltage *V* along the *y*-direction between two ends of the Pt strip was measured when temperature difference Δ*T* was applied in the *x*-direction. To magnetize the ferrite, an external magnetic field *H* was also applied in the *x*-direction. (b) Measured voltage *V* as a function of external magnetic field *H* when Δ*T* = 1.1K was applied to the sample.

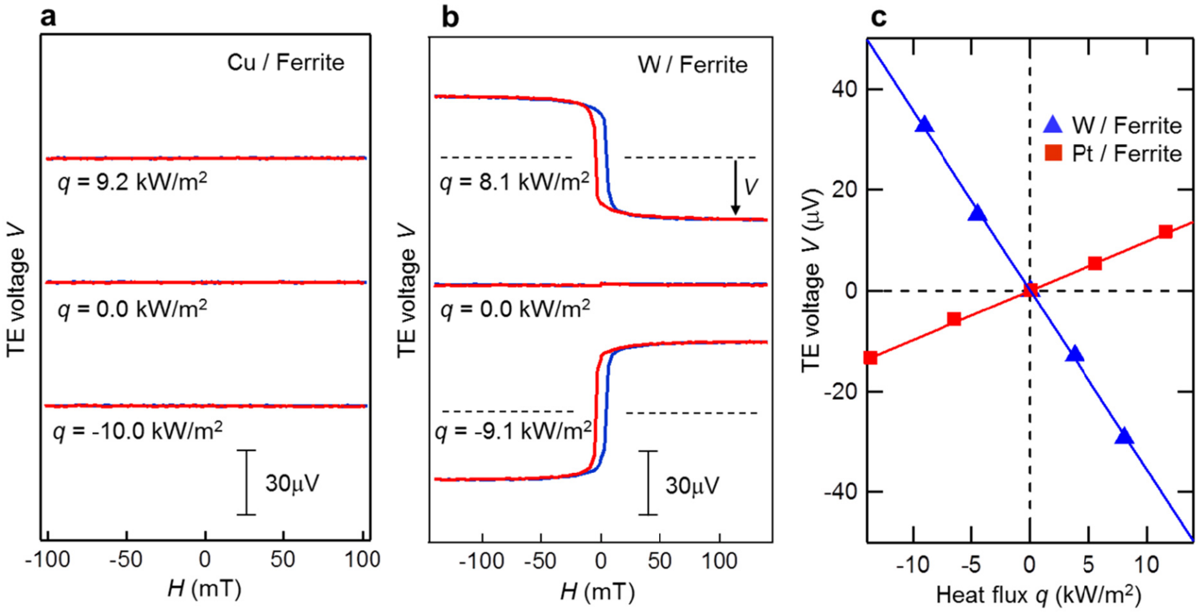


**Figure S2 | SSE-based TE sheets with different metallic films.** (a) TE voltage *V* from a Cu/$Ni_{0.2}Zn_{0.3}Fe_{2.5}O_4$/polyimide sample as a function of an external magnetic field *H*, measured when the heat flux *q* was applied across the sample. The voltage was negligibly small, due to the small ISHE in Cu. (b) TE voltage *V* from a W/$Ni_{0.2}Zn_{0.3}Fe_{2.5}O_4$/polyimide sample obtained with the same measurement setup. The voltage signal has a sign opposite to that of the Pt/$Ni_{0.2}Zn_{0.3}Fe_{2.5}O_4$ sample

because the spin-Hall angle of W has the opposite sign to that of Pt. (c) TE voltage from the TE sheet W/$Ni_{0.2}Zn_{0.3}Fe_{2.5}O_4$ compared with that from Pt/$Ni_{0.2}Zn_{0.3}Fe_{2.5}O_4$ as a function of $q$. According to the fitting with the solid line, the heat-flow sensitivity of W/$Ni_{0.2}Zn_{0.3}Fe_{2.5}O_4$ was evaluated to be $V/q$ = 3.55 nV/(W/m$^2$), which is more than 3 times larger than that of Pt/$Ni_{0.2}Zn_{0.3}Fe_{2.5}O_4$.
